# Whole-exome sequencing and genome-wide methylation analyses identify novel disease associated mutations and methylation patterns in idiopathic hypereosinophilic syndrome

**DOI:** 10.18632/oncotarget.5845

**Published:** 2015-10-19

**Authors:** Christen Lykkegaard Andersen, Helene Myrtue Nielsen, Lasse Sommer Kristensen, Alexandra Søgaard, Jonas Vikeså, Lars Jønson, Finn Cilius Nielsen, Hans Hasselbalch, Ole Weis Bjerrum, Vasu Punj, Kirsten Grønbæk

**Affiliations:** ^1^ Department of Hematology, Rigshospitalet, Copenhagen University Hospital, Copenhagen, Denmark; ^2^ Department of Hematology, Roskilde University Hospital, Roskilde, Denmark; ^3^ Department of Clinical Biochemistry, Rigshospitalet, Copenhagen University Hospital, Copenhagen, Denmark; ^4^ Keck School of Medicine, University of Southern California, Los Angeles, California, USA

**Keywords:** eosinophilia, idiopathic hypereosinophilic syndrome, diagnosis, next-generation sequencing, genome-wide DNA methylation analysis

## Abstract

A thorough understanding of the idiopathic hypereosinophilic syndrome (IHES) and further optimization of diagnostic work-up procedures are warranted. We analyzed purified eosinophils from patients with IHES by next-generation whole-exome sequencing and compared DNA methylation profiles from reactive eosinophilic conditions to known clonal and suspected clonal eosinophilia. Somatic missense mutations in cancer-related genes were detected in three IHES patients. These included the spliceosome gene *PUF60* and the cadherin gene *CDH17*. Furthermore, reactive eosinophilia samples could be differentiated from known- and suspected clonal eosinophilia samples based on 285 differentially methylated CpG sites corresponding to 128 differentially methylated genes. Using Ingenuity pathway analysis, we found that differentially methylated genes were highly enriched in functional pathways such as cancer, cell death and survival, and hematological disease. Our data show that a subset of IHES may be of clonal origin not related to the classical molecular aberrations of *FGFR, PDGFRA/B,* or T-cells, and that the initiating hits could be point mutations in a variety of genes, including spliceosome mutations or hypermethylated tumor suppressor genes. In addition, we identified a DNA methylation signature that is relevant for distinguishing clonal and suspected clonal eosinophilia from reactive eosinophilia *per se*, which may be useful in daily clinical work.

## INTRODUCTION

In healthy individuals, eosinophilic granulocytes (eosinophils) constitute less than five percent of all white blood cells [1], and in clinical practice blood eosinophilia is defined as an eosinophil count ≥ 0.5×10^9^/l. Eosinophilia arises either as an intrinsic, clonal disorder or in the majority of cases, secondary to extrinsic conditions, so-called reactive eosinophilia [2–4]. A plethora of distinct disease entities with concomitant eosinophilia has been known for many years, whereas the primary eosinophilic conditions were only introduced in 1968 [1, 5, 6]. Advances in cytogenetic and, in particular, molecular techniques have identified specific lymphoid and myeloid neoplasms with eosinophilia, hereby categorizing clonal markers in these entities [3, 4, 7]. In addition, chromosomal aberrations of *FGFR* and *PDGFRB*, point mutations in *PDFGRA*, and T-cell clonality have been identified in rare cases of primary eosinophilia. This leaves a very small subgroup of patients with *idiopathic hypereosinophilia (IHE)* and *idiopathic hypereosinophilic syndrome (IHES),* [3, 4, 7, 8] where clonality is often suspected, however, neither genomic aberrations nor other triggering stimuli can be demonstrated.

In clinical practice, the diagnostic workup of patients presenting with eosinophilia is particularly challenging and resource intensive. For patients in whom reactive causes are eventually excluded, only a few definite cytogenetic or molecular markers exist. Thus, a thorough understanding of these heterogeneous conditions and further optimization of the diagnostic work-up procedures are highly warranted.

Recently, whole-exome sequencing has proven of great value in the identification of novel point mutations in myeloproliferative neoplasms [9, 10] and DNA methylation profiling has identified clinically distinct - and prognostic - subgroups in myeloproliferative neoplasms [11], myelodysplastic syndrome (MDS) and acute myeloid leukemia (AML) [12].

Our main hypothesis is that some subtypes of IHES are indeed clonal disorders, thus in the current study, our aims were two-fold. First, to uncover candidate disease associated mutations by analyzing purified eosinophils from patients diagnosed with IHES by next-generation whole-exome sequencing (NGS). Second, to uncover DNA methylation profiles that distinguish clonal and suspected clonal IHES from reactive eosinophilic conditions, based on the hypothesis that IHES are either driven by DNA methylation changes of growth associated genes, or represents a clonal disorder with a yet undiscovered stimulus.

## RESULTS

### Patients

The included patients with known clonal and suspected clonal eosinophilia were females (*n* = 3) and males (*n* = 6) of varying age, and with both newly diagnosed and longstanding disease (range: 0–263 weeks). Four patients received cytoreductive therapy at time of blood sampling (prednisolone, busulfan, hydroxyurea and mycophenolate mofetil), whereas the other 5 were under observation. Peripheral eosinophil levels ranged from 0.8–5.8×10^9^/L where the highest concentration was noted in a newly diagnosed IHES patient receiving prednisolone. Further details regarding cytogenetic and molecular analyses performed at diagnosis are shown in Table 1.

**Table 1:** Characteristics of included patients

Time of diagnosis										Time of blood sampling									
Patient ID	Diagnosis	Gender	Allergies	Cytogenetics	*JAK2* V617F	c-KIT status	TCR-analysis	FIP1L1-PDGFRA	BCR/ABL	Age	Weeks since diagnosis	Hemoglobin (mmol/L)	Leukocytes (10^9^/L)	Eosinophils (10^9^/L)	Platelets (10^9^/L)	LDH (U/L)	CRP (mg/L)	IgE (10^3^IU/L)	Immunosuppressive therapy
S1	IHES	F	Tramadol	46,XX(25)	N/P	N/P	Inconclusive	Negative	N/P	70	164	9.0	9.5	1.3	228	246	37	N/P	Prednisolone, Bulsulfan
S2	IHES	M	None	45,XY, der(13;14)(q10;q10)c(25)	Negative	N/P	Negative	Negative	N/P	25	97	6.6	9.1	5.1	143	353	9	N/P	Hydroxyurea, Prednisolone
S3	IHES	F	None	46,XX(25)	N/P	N/P	Negative	Negative	N/P	56	0	6.3	11.6	5.8	471	340	4	N/P	Prednisolone
S4	IHES	M	Digoxin	N/P	N/P	N/P	Negative	Negative	N/P	62	12	7.7	12.4	2.0	260	N/P	3	2320	None
S5	IHES	F	None	N/P	N/P	N/P	Negative	Negative	N/P	39	194	8.7	12.5	1.2	248	175	<1	N/P	Prednisolone, Mycophenolate mofetil, Hydroxyurea
S6	SM	M	None	46, XY (27)	N/P	Positive	Negative	Negative	N/P	71	263	7.6	8.6	1.7	318	104	5	3	None
S7	CMML	M	None	46, XY (25)	Negative	N/P	N/P	Negative	Negative	81	10	6.9	21.9	1.3	151	235	61	2036	None
S8	IHES	M	None	46, XY (25)	Negative	N/P	Negative	Negative	N/P	61	2	8.2	21.4	3.8	355	250	1.1	N/P	None
S9	IHES	M	None	45,X,-Y(7)/46,XY(18)	Negative	N/P	N/P	Negative	Negative	68	25	8.7	10.4	0.8	342	101	<1	N/P	None
R1	Eosinophil fascitis	M	None	N/P	N/P	N/P	N/P	N/P	N/P	60	0	9.2	8.1	0.9	287	195	22	N/P	None
R2	Schistosomiasis	M	None	N/P	N/P	N/P	N/P	N/P	N/P	41	1	8.2	16.5	9.2	381	283	18	8810	None
R3	Ficulin-3 deficiency	M	None	N/P	N/P	N/P	N/P	N/P	N/P	35	607	7.0	12.3	1.0	376	162	11	N/P	None

IHES, idiopathic hypereosinophilic syndrome; SM, systemic mastocytosis; CMML, chronic myelomonocytic leukemia; N/P, not performed; TCR, T-cell receptor

### Somatic mutations are present in a subset of IHES patients

Whole-exome NGS was possible to perform for five of the seven IHES patients (samples S1-S5). Of these, somatic mutations (present in the patients' eosinophils, but not lymphocytes) were detected in three of five patients considered to have IHES. These are summarized in Table 2. No mutations were detected in two patients (S3 and S4). Somatic missense mutations in the coding regions of genes were detected in patients S1 (*n* = 3), and S2 (*n* = 1). Interestingly, most of these mutations were located in cancer-related genes, although these patients did not have any (yet) recognized, underlying cancer.

**Table 2:** Overview of mutations detected by whole-exome sequencing of IHES patient samples

Patient ID	Gene	Chromosomal region	Mutation	Consequence	Transcript ID	SIFT Function Prediction	Zygosity	dbSNP build 141
S1	*LMLN*	3:197,729,933	c.1280G > T	p.R427L	NM_001136049.2	Alter function	Heterozygous	Not present
S1	*CDH17*	8: 95,143,139	c.2249G > A	p.G750D	NM_001144663.1	Alter function	Heterozygous	Not present
S1	*PUF60*	8: 144,900,231	c.438C > G	p.S146R	NM_001271097.1	Alter function	Heterozygous	Not present
S2	*AQP12A*	2: 241,621,869	c.386C > T	p.T129M	NM_001102467.1	Tolerated	Heterozygous	rs74882485
S5	*PCSK1*	5: 95,768,842	c.-96C > T	5′UTR	NM_000439.4	N/A	Heterozygous	rs35753085

In patient S1 we identified a total of 3 point mutations, which may potentially influence gene function. The first is a missense mutation that causes S146R in the spliceosome gene *PUF60*. PUF60 is involved in a ribonucleoprotein (RNP) complex and modulates alternative splicing of several mRNAs by binding to the pyrimidine tract and 3′-splice site regions of pre-mRNA [17]. In addition, it is involved in regulation of MYC transcription [18], and it is known that certain PUF60 splice variants are overexpressed in ovarian and gastric cancer [19–21]. The second mutated gene in S1 was *CDH17*, which has been shown to promote tumorigenesis and metastasis through Wnt-signaling [22] and the third, the *LMLN* gene, which encodes a zinc-metallopeptidase, has not previously been implicated in cancer. In patient S2 we observed a T129M in *AQP12A*, which is not predicted to alter protein function, and this gene has not been implicated in cancer. In patient S5 we detected a somatic mutation in the 5′ UTR of the *PCSK1* gene. Germline mutations in this gene have been implicated in monogenic obesity, however, promoter hypermethylation have also been shown in malignant melanoma [23].

**Figure 1:**
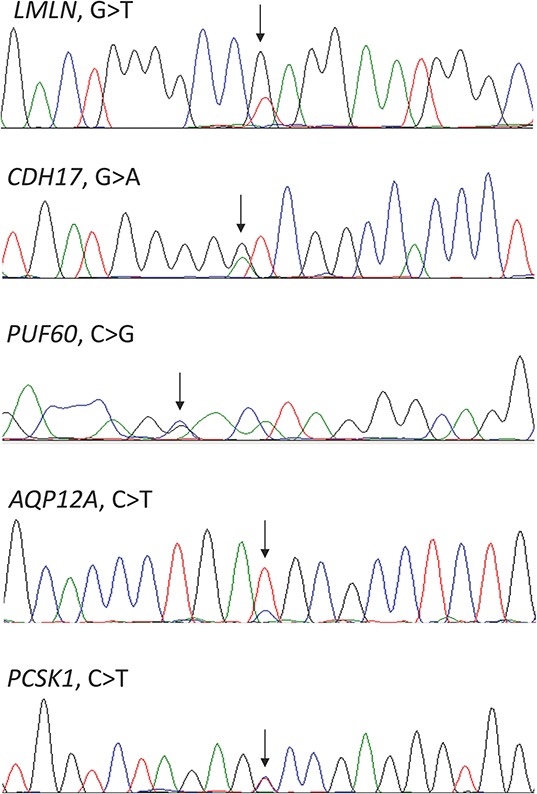
Confirmation by Sanger sequencing of the mutations detected by whole-exome sequencing Arrows indicating the positions of the mutations.

All of these mutations could be confirmed by Sanger sequencing in archived bone marrow samples from time of diagnosis (Figure 1). This is an important finding as the mutations could, otherwise, have been therapy-induced, and thus not part of the natural history of the disease.

### DNA methylation distinguish clonal and suspected clonal eosinophilia from reactive eosinophilia

**Figure 2:**
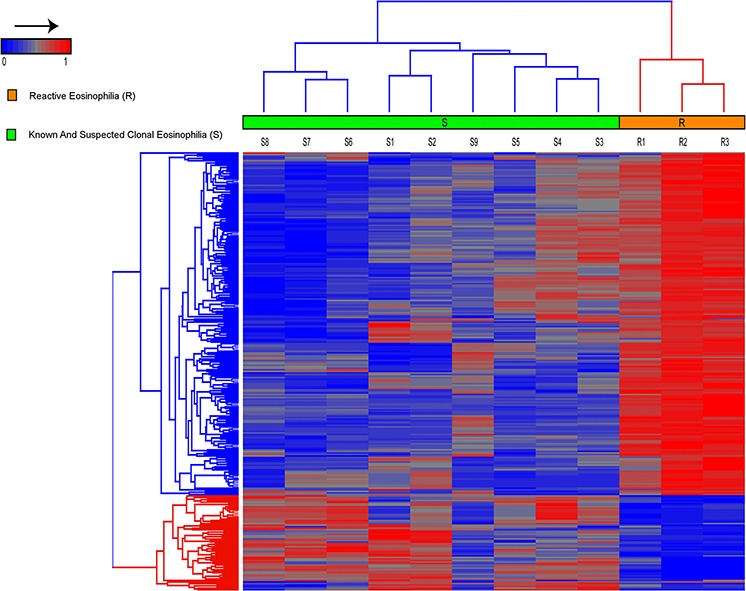
Hierarchical clustering, using Euclidean distance and complete linkage of 285 probes, show differential methylation between patients with known and suspected clonal eosinophilia (S1–S9) versus patients with reactive eosinophilia (R1–R3) In general, an overall hypomethylation distinguished S - from R-samples, and in addition S-samples were characterized by a number of specific hypermethylated CpG sites.

The Illumina 450K Infinium platform was used to identify differentially methylated CpG sites between samples S1-S9 and R1-R3. Reactive eosinophilia samples (R1-R3) could be differentiated from known and suspected clonal eosinophilia samples (S1-S9) based on 285 differentially methylated probes/CpG sites with a Δβ ≥ 0.2 (Figure 2). From these, we identified a 128 gene methylation signature that could differentiate between reactive eosinophilia and known - and suspected - clonal eosinophilia (Supplementary Table 2). When comparing differentially methylated regions, S samples showed a general hypomethylation relative to R samples. Similarly, an overview of these 285 probes in healthy controls (C) suggests that a majority of the probes (80%) were constitutively hypermethylated with average beta values > 0.2 (data not shown).

**Figure 3:**
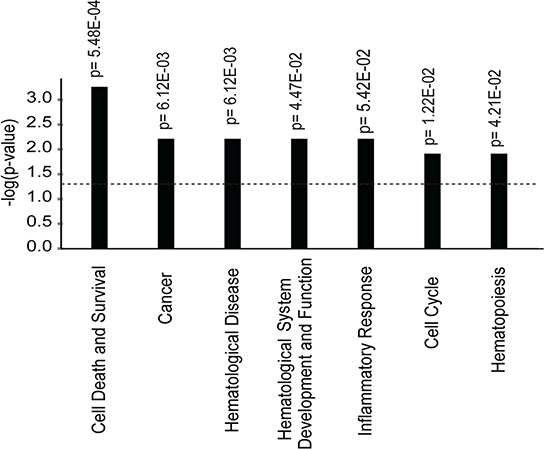
Functional gene enrichment based on the 128 genes corresponding to 285 differentially methylated probes that distinguish patients with known and suspected clonal eosinophilia from patients with reactive eosinophilia Functional pathways, with significantly enriched gene sets, are ranked according to their *p*-values shown on Y-axis.

*Mir886, GSTM5, TNXB, ZADH2, LGR6, HLA-C, HLA-DRB1, S100A13, HIVEP3* had the highest number of differentially methylated probes annotated. Using Ingenuity pathway analysis, we found that differentially methylated genes were highly enriched in functional pathways such as cancer, cell death and survival, hematological disease, and inflammatory response (Figure 3). To further understand the implications of these differentially methylated signature genes, we identified a set of 31 genes (Supplementary Table 3), which overlap with known cancer associated genes available in public databases (http://www.broadinstitute.org/; TS Gen - http://bioinfo.mc.vanderbilt.edu/TSGene/). In order to verify the genome-wide DNA methylation analysis we used pyrosequencing to obtain quantitative DNA methylation levels at differentially methylated CpG sites (Supplementary Figure 1). The DNA methylation status of two differentially methylated probes was verified as the methylation status obtained from the Illumina array and pyrosequencing correlated highly (R^2^ > 0.9 for both assays).

## DISCUSSION

With the revision of the 2008 World Health Organization (WHO) classification of myeloid neoplasms, a new category entitled *Myeloid and lymphoid neoplasms with eosinophilia and abnormalities of platelet-derived growth factor receptor alpha (PDGFRA), platelet derived growth factor receptor beta (PDGFRB), or fibroblast growth factor receptor 1 (FGFR1)* was introduced [24]. This new entity acknowledged the last two decades' discoveries of molecularly well-defined clonal eosinophilic conditions. Within recent years multiple point mutations have been detected in the genes encoding the mRNA splicing machinery (spliceosome) in hematological malignancies including MDS, AML and chronic lymphocytic leukemia [25] as well as in several solid tumors [26]. However, mutations within the spliceosome have not previously been reported for clonal eosinophilia, possibly due to a lack of whole-exome studies performed for this rare disease. Here we, for the first time, report a somatic spliceosome mutation in a patient with eosinophilia (Patient S1, Table 2), with no other underlying myeloproliferative disorder. This mutation was discovered in a gene, *PUF60*, which is involved in multiple cellular functions. Firstly, it facilitates recognition of 3′ splice sites in conjunction with *U2AF65* [17, 20]. PUF60 has also been reported to be part of a complex at the *MYC* promoter, which represses *MYC* transcription [18]. In addition, it was recently shown that *PUF60* is part of a long non-coding RNA (lncRNA)-associated ribonucleoprotein (RNP) complex, which regulates breast cancer metastasis through modulation of a translational regulatory lncRNA (treRNA), which suppresses the translation of E-cadherin (*CDH1*) mRNA [27]. Interestingly, *PUF60* has previously been reported to be mutated in several cancers, at very low frequencies (http://cancer.sanger.ac.uk/cancergenome/projects/cosmic/) and it has been implicated in ovarian and gastric cancer [19, 21]. According to the SIFT algorithm, the S146R substitution is predicted to disrupt the normal PUF60 activity, however, functional studies are obviously warranted to unravel the effects of this particular alteration. Since a point mutation in the well-known cancer associated gene, *CDH17*, was also discovered within the same patient sample in the current study, it could be speculated that these two mutations cooperate in promoting the oncogenic potential of the clone. Finally, we also discovered a somatic mutation in *LMLN*, a gene encoding a zinc-metallopeptidase, in this patient. Metallopeptidases are known to play a role in cancer, however, it is not known if *LMLN* is directly implicated in tumorigenesis. This particular patient was followed for 5 years at our institution with aggressive IHES. Her disease responded to some degree to prednisolone, while she had been unsuccessfully treated with hydroxyurea, interferon, imatinib, dasatinib, alemtuzumab, cyclophosphamide and mycophenolate mofetil. She succumbed from pneumonia in 2013.

A somatic mutation in the *AQP12A* gene was observed in patient S2. However, this mutation has also been reported in dbSNP (rs74882485) (minor allele frequency [MAF]: 2.811%), and is predicted to be tolerated by SIFT. Therefore, this is most likely a passenger mutation. We did not detect mutations in the coding regions of any candidate driver genes in this patient, and it is likely that this clone is driven by other molecular mechanisms. It should be noted that all none-deleterious mutations were filtered out and a manual inspection of all possible mutations is likely to prevent reporting of false-positive mutation calls.

Lastly, in patient S5 we detected a mutation in the 5′ UTR of the *PCSK1* gene that encodes an enzyme which functions in the proteolytic activation of polypeptide hormones and neuropeptide precursors. *PCSK1* is mainly implicated in obesity [28], but promoter hypermethylation has been reported in cancer, mainly in malignant melanoma. The mutation we detected locates to the 5′ UTR, and could potentially affect gene regulation, however as it does not change the coding sequence, it is less likely to be of functional significance. In addition, this mutation has been reported in dbSNP (rs35753085) (minor allele frequency [MAF]: 0.504%). For patients S2 and S5 we were unable to relate these observations to potential adverse clinical outcomes.

Thus, overall, we found somatic mutations in three out of five of the IHES patient samples. However, since two of the patients did not carry any somatic mutations in the coding regions and the mutations detected in two other patients may be passenger mutations, it is likely that other molecular changes such as mutations in noncoding regions and epigenetic aberrations may also play an important role in IHES. Accordingly, we show, for the first time that suspected clonal - and reactive eosinophilia may be distinguished by differentially methylated regions using state-of-the-art technology for genome-wide DNA methylation analysis. In total, 285 probes corresponding to 128 unique genes were differentially methylated in samples from patients with known and suspected clonal eosinophilia compared to patients with reactive eosinophilia, respectively (Figure 2, Supplementary Table 2).

IHES cases showed general hypomethylation with local hypermethylation when compared to reactive eosinophilia (Figure 2) and control samples (data not shown), and several of the differentially methylated genes have previously been linked to cancer (Supplementary Table 2). For example, chromosome 5q31 harbors *MIR886* (also known as *VTRNA2–1* and *nc886*), which encodes a non-coding RNA that is found monoallelically methylated in 75% of healthy individuals, whereas the remaining 25% are unmethylated [29, 30]. miR886 has been considered a tumor suppressor as 5q31 often is lost in several tumors [30, 31] and, in addition, gain of methylation and concurrent decreased gene expression has been found to predict outcome in patients with AML [30]. However, both gain and loss of methylation of *MIR886* have been implicated in different cancer types including breast-, colon-, bladder-, and lung cancer [29]. In our study, five out of nine patients with anticipated and known clonal eosinophilia were unmethylated at *MIR886* while the remaining four were 50% methylated. The three samples with reactive eosinophilia were all 50% methylated.

The promoter region of *GSTM5* was hypermethylated in patients with anticipated and known clonal eosinophilia (Supplementary Table 2). In healthy cells, GSTM5 detoxifies endogenous compounds, including carcinogens, and therefore, epigenetic silencing of *GSTM5* may increase an individual's sensitivity to carcinogens and other toxic compounds. The promoter region of *S100A13* was hypomethylated in patients with anticipated and known clonal eosinophilia. S100A13 comprises, together with IL1alpha, a complex which has been found to have an essential role in cell proliferation, differentiation, and angiogenesis [32]. Interestingly, inhibition of the IL1alpha-S100A13 complex has been suggested to be an effective strategy to inhibit uncontrolled cell division of a broad range of different cancer types [32].

By the use of public databases additional genes were found to be known cancer associated thereby further supporting that the identified methylation signature genes may be important in blood eosinophilia of non-reactive origin (Supplementary Table 3). Accordingly, pathway analysis showed that differentially methylated genes were preferentially involved in cancer, cell death and survival, hematological disease, and inflammatory response (Figure 3), indicating the presence of an underlying DNA methylation driven clonal evolution in IHES that is distinguishable from a reactive condition. Whether the differential methylation observed has functional consequences is a subject for further investigation.

Nevertheless, our data suggest that identification of differentially methylated regions may be useful markers for differentiating clonal – and suspected clonal eosinophilia from reactive eosinophilia, irrespective of function. However, further studies in larger patient cohorts are obviously needed to confirm and extend our findings, hopefully identifying a panel of useful biomarkers with clinical potential.

In conclusion, our data indicate that a subset of IHES may be of clonal origin unrelated to molecular aberrations of *FGFR, PDGFRB, PDFGRA*, or T-cells. The initiating hits could be point mutations in a variety of genes, including spliceosome mutations and a number of genes previously associated with cancers. In addition, we show that aberrant DNA methylation patterns can distinguish clonal and suspected clonal eosinophilia from reactive eosinophilia, which may be very useful in daily clinical work. Whether or not epigenetic events could be disease initiating or drive the eosinophilic clone is an intriguing subject for further investigation.

## MATERIALS AND METHODS

### Patients

We included patients with eosinophilia of known clonal origin (SM [systemic mastocytosis], *n* = 1; CMML [chronic myelomonocytic leukemia], *n* = 1) or suspected clonal origin (IHES [*n* = 7]) from the outpatients clinic at department of hematology, Rigshospitalet (RH). These patients were designated S1-S9 (Table 1). We excluded potential candidates if they did not exhibit blood eosinophilia (≥ 0.5×10^9^/L) due to medical intervention or received targeted therapy (i.e. imatinib). Patients with reactive eosinophilia were included from department of rheumatology, RH (one patient with eosinophil fasciitis) and department of infectious medicine, RH (one patient with immunodeficiency associated with FCN3 mutation and Ficolin-3 deficiency [13] and one patient with schistosomiasis infection). These patients were designated R1-R3 (Table 1). Healthy controls (*n* = 6, M/F= 2/4) (C samples) with no known disease, allergies or concurrent therapies were also included in the methylation analyses. The study was approved by The Danish Data Protection Agency (journal no: 2011–41-5821), and The National Committee on Health Research Ethics (journal no: H-2–2011-010). All patients and volunteers gave written informed consent before participation.

### Purification of eosinophils and lymphocytes

Fifty mL of freshly drawn heparinized venous blood from patients with known clonal and suspected clonal eosinophilia (samples S1-S9), and reactive eosinophilia (samples R1-R3) and healthy controls was diluted with an equal amount of cooled phosphate buffered saline. Peripheral blood granulocytes were isolated after Ficoll Paque PLUS (GE Healthcare^®^) gradient separation. Eosinophils were sorted from the granulocyte layer by the EasySep™ Human Eosinophil Enrichment Kit on a RoboSep device (StemCell Technologies^®^). The purity of the sorted eosinophils was > 98%. From the mononuclear layer, we isolated total lymphocytes by the use of the EasySep™ Human Whole Blood Lymphoid Positive Selection Kit. Subsequent DNA purification of both eosinophils and lymphocytes was performed by the use of Qiagen^®^ DNeasy according to the manufacturer's instructions.

### Exome sequencing and data analysis of DNA isolated from eosinophils and lymphocytes

Exome enrichment was performed using SureSelect All Exon kits v4 (Agilent Technologies), which captures 50 Mb of coding sequence, and sequencing was performed on either Illumina Genome Analyzer IIx or the HiSeq 2500 platform. In brief, 3 μg of genomic DNA isolated from eosinophils or lymphocytes was fractionated on a Covaris S2 to an average size of 200 base pairs. Trimming, 3′ adenylation and ligation of Illumina TruSeq DNA adaptors were performed on SPRI-TE nucleic acid extractor using the SPRI works Fragment Library Cartridges I (Beckman Coulter) with a size selection of 200–400 base pairs. Sequencing was either performed on the Genome Analyzer IIx as paired end (PE) 2x76 bases sequencing, on which a single exome was sequenced per lane on the flow cell resulting in 55–60 M PE reads/sample, or on the HiSeq 2500 as PE 2x101 bases sequencing where 4 samples were multiplexed per lane, resulting in approximately 59 M PE reads/sample. Sequencing data were processed to FASTQ files using CASAVA-1.8.2, and the data were further processed using CLC Genomics Server v5.5 software and Ingenuity Variant Analysis. The bioinformatics workflow is described in detail in Supplementary Appendix 1.

### Sanger sequencing of diagnostic samples

To assess if mutations detected by NGS (please refer to “Results”) were also present in diagnostic bone marrow samples from the time of diagnosis, PCR primers flanking the identified mutations were designed (See Supplementary Table 1). The PCR contained 20–50 ng of genomic DNA, 1 x PyroMark PCR master mix (QIAGEN, Hilden, Germany), 1 x CoralLoad Concentrate (QIAGEN, Hilden, Germany), and 200 nM of each primer in a final volume of 25 μl. The PCR program was initiated with a denaturation step of 15 min at 95°C followed by 45 cycles of 20 sec at 95°C, 30 sec at 60°C, and 30 sec at 72°C. The final extension was performed for 10 min at 72°C. The PCR products were confirmed to be of the correct size by gel electrophoresis and subsequently Sanger sequenced in either forward or reverse direction by the service of Eurofins^®^.

### Genome wide DNA methylation

Genome wide DNA methylation profiling was performed on 450K Infinium arrays (Illumina Inc.). This platform comprehensively interrogates the DNA methylation status of more than 480,000 CpGs in the human genome corresponding to 99% of all NCBI RefSeq genes, which include CpGs in the promoters, enhancers, and gene bodies as well as CpGs located outside coding regions. In addition, probes have been mapped to CpG islands as well as shores and shelves of CpG islands (http://www.Illumina.com). The Infinium DNA methylation assay was performed at Genomic Core at USC Epigenome Center, Los Angeles and β-values representative of level of methylation were calculated as described previously [14].

### Bioinformatics analysis of differential methylation

Infinium probes that failed in any of the samples or showed a detection *p* > 0.05 over the background signal were excluded from the analysis. Further, probes containing a single nucleotide polymorphism (SNP) or containing a repetitive element within five base pairs of targeted CpG sites were excluded. Two additional filters were used in order to obtain the differential methylation pattern associated with the known clonal and suspected clonal eosinophilic patients (samples S1-S9). Firstly; probes which showed β≤ 0.05 in 50% of the samples within a group are likely non-informative probes and were excluded. Secondly, constitutively methylated probes in more than 80% of healthy control samples (β ≥ 0.5) were excluded. Given that the main clinical challenge is to separate clonal and suspected clonal (S) from reactive (R) eosinophilia, we aimed at identifying biomarkers for this purpose. Accordingly, control samples (C) were not included in the subsequent methylation analyses between R and S samples. For differential methylation analysis various criteria have been described in the literature; to identify the CpG sites showing differential methylation, we used a mean β-value difference of ± 0.2 between R - and S groups. Hierarchical clustering, data visualization as well as statistical analysis were performed in R software environment except as noted (http://www.r-project.org). Genes which were differentially methylated between reactive (R) - and known clonal and suspected clonal (S) groups were functionally analyzed in the context of gene ontology and molecular networks by using Ingenuity pathway software (IPA; http://www.ingenuity.com) as detailed previously [15]. Differentially methylated genes were categorized into various functional groups using a threshold *P* < 0.05 and mapped to genetic networks.

### Validation of genome-wide DNA methylation analysis by pyrosequencing

Pyrosequencing was used to obtain quantitative DNA methylation data [16]. In brief, 500 ng of genomic DNA were sodium bisulfite treated using the EZ DNA methylation^TM^ Kit (Zymo Research) according to the manufacturer's instructions. The PCR contained 20 ng of sodium bisulfite treated DNA, 1 x PyroMark PCR master mix (QIAGEN, Hilden, Germany), 1 x CoralLoad Concentrate (QIAGEN, Hilden, Germany), and 200 nM of each primer in a final volume of 25 μl. The PCR program was initiated with a denaturation step of 15 min at 95°C followed by 45 cycles of 30 sec at 95°C, 30 sec at 58°C, and 30 sec at 72°C. The final extension was performed for 10 min at 72°C. Primer sequences are given in Supplementary Table 1.

## SUPPLEMENTARY DATA




