# Supplementary material for: Whole-exome sequencing and genome-wide methylation analyses identify novel disease associated mutations and methylation patterns in idiopathic hypereosinophilic syndrome
Source: Oncotarget. 2015 Oct 19;6(38):40588–97. doi: 10.18632/oncotarget.5845 (PMC4747354; doi:10.18632/oncotarget.5845)
Supplement: Supplementary file 1 [file oncotarget-06-40588-s001.pdf]

SUPPLEMENTARY DATA

Supplementary appendix 1

FASTQ files were further processed using the CLC Genomics Server v5.5 software. Reads were trimmed to remove bases with a phred-score > 0.01, all ambiguous reads, the last 3′ base, reads with a length below 30 and finally all duplicate reads were merged into one. Mapping was performed using default settings except length fraction and similarity fraction were set to 0.95. Variants were called using Probabilistic Variant Detection (minimum coverage 10, variant probability > 95.0). To identify variants specific for eosinophils, the eosinophil variants were filtered against the lymphocytes mapped reads. The following filter criteria were applied: Minimum read count > 3, frequency > 30%, forward reverse balance > 0.2, control frequency < 10% and control coverage > 9. We allowed a control frequency < 10% since eosinophil sample preparation can contain small contaminations from lymphocytes. Subsequently, the lists of variants were imported into Ingenuity Variant Analysis for further filtering. Here, the variants were filtered to remove variants in the top 0.2% most exonically variable 100base windows in healthy public genomes and variants in the top 1% most exonically variable genes in healthy public genomes (1000 Genomes). Next, only variants predicted to be potentially deleterious were included, being variants with frameshift, in-frame indel, nonsense mutations, missense or likely splice site loss (up to 10 bases into intronic region). Finally, variants passing all these criteria were manually inspected in CLC Genomic Workbench selecting only true *de novo* mutations.

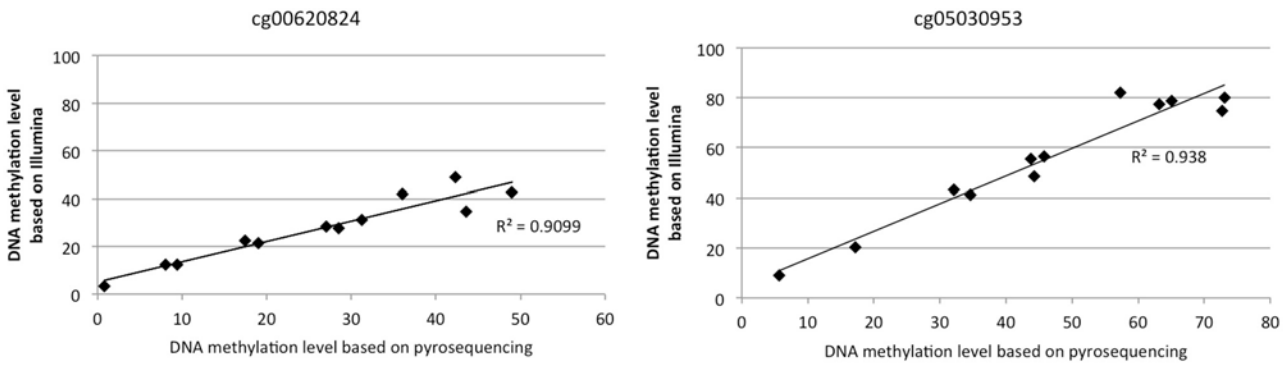

Supplementary Figure S1: Validation of the DNA methylation data obtained using Illumina's 450K BeadChip array. The DNA methylation level of two CpG sites was verified using pyrosequencing and for each CpG site the verified DNA methylation level correlated highly between pyrosequencing and Illuminas 450K BeadChip array with a coefficient of determination higher than 0.91.

Supplementary Table S1: Assays used for Sanger sequencing for confirmation of mutations in diagnostic samples and validating the genome-wide DNA methylation analysis, respectively

Gene	Primer sequence	Genomic location
CDH17	F: 5′TCATGCCCGACTGTCTACCAG<br>R: 5′AGAAACAATGCCTTCCAAGGGTG	chr8:95, 143, 111–95, 143, 216
PUF60	F: 5′CTGGGTTGACCGGTCTTTCC<br>R: 5′TCTATGATGGGCTGGGCCTG	chr8:144, 900, 202–144, 900, 286
LMLN	F: 5′AAGCAAATTACAGCATGGCTGAG<br>R: 5′CTTTTGTCTCTGCTGATCAATCCA	chr3:197, 729, 894–197, 729, 997
AQP12A	F: 5′TCCCTGCAGAAGTTCCTCATGGC<br>R: 5′ TGCAGGTCACTGAGCTCCCA	chr2:241, 621, 833–241, 621, 966
PCSK1	F: 5′ AGACCGAAGCGCTTCACTGA<br>R: 5′ AGCAAGATAGGAGAAAAGCCAGA	chr5:95, 768, 795–95, 768, 941

Probe no.	Location of probes	Primer sequence*	Pyrosequencing primer	No. of CpG sites analyzed	Genomic location
cg00620824(*HLA-C*)	1500TSS	F: 5′-gaagTagggtttgttaTtgtTtattgTa<br>R: 5′-AtccaaataaaatatAcacactActtaAat	F: 5′-ggtttgttaTtgtTtattgTaaT	1	chr6_mann_hap4:2, 587, 634–2, 587, 793
cg05030953(*HLA-C*)	1500TSS	F: 5′aaggagTagaggaaagaattTtaaagTagt<br>R: 5′-aAcccattaAttttaaAAcaAtcacaca	F: 5′-agTTtggTagggggt	1	chr6_mcf_hap5:2, 621, 479–2, 621, 583

* In the DNA methylation assays, bisulfite converted non-CpG cytosines are indicated as T/A on the sense/antisense strand, respectively

Supplementary Table S2: The 285 probes corresponding to 128 unique genes that were differentially methylated in samples from patients with known and suspected clonal eosinophilia (S samples) and patients with reactive eosinophilia (R samples). The table is sorted by gene name.

Supplementary Table S3: Differentially methylated oncogenic signature genes in our dataset

Gene name	Genomic annotation*	CpG neighborhood	Comment
TSKS	Body	Island	hypermethylation in S
SPTLC2	Body	Open	hypermethylation in S
C1orf109	Promoter	Shore	hypermethylation in S
HCG9	Promoter	Shore	hypermethylation in S
CREB3L2	3'UTR	Open	hypermethylation in S
PM20D1	Promoter	Island	hypermethylation in S
GSTM1	Promoter	Shore	hypermethylation in S
RPS6KA2	Body	Open	hypermethylation in S
PF4	Promoter	Island	hypermethylation in R
TRIM41	Promoter	Shore	hypermethylation in R
PTH1R	Body	Shelf	hypermethylation in R
LRRC61	Promoter	Island	hypermethylation in R
ANKRD53	Promoter	Island	hypermethylation in R
TP53I13	3'UTR	Island	hypermethylation in R
SLC17A3	Promoter	Open	hypermethylation in R
EPS8L1	Body	Island	hypermethylation in R
SPTBN1	Body	Shore	hypermethylation in R
C3orf32	Promoter	Open	hypermethylation in R
KIAA1274	Promoter	Open	hypermethylation in R
SERHL	Promoter	Island	hypermethylation in R
KCNK3	Body	Island	hypermethylation in R
OXT	Promoter	Island	hypermethylation in R
TCF7L2	Body	Shore	hypermethylation in R
RAI1	Promoter	Island	hypermethylation in R
HSD17B1	Body	Island	hypermethylation in R
BTG2	Promoter	Shore	hypermethylation in R
FN3K	Body	Island	hypermethylation in R
TNXB	Body	Island	hypermethylation in R
CRIP2	Body	Island	hypermethylation in R
SLC39A4	Body	Island	hypermethylation in R
C3	Promoter	Open	hypermethylation in R

[*] As per UCSC genome Table. Promoter is 2000bp across TSS
