# Supplementary material for: Whole-exome sequencing and genome-wide methylation analyses identify novel disease associated mutations and methylation patterns in idiopathic hypereosinophilic syndrome
Source: Oncotarget. 2015 Oct 19;6(38):40588–97. doi: 10.18632/oncotarget.5845 (PMC4747354; doi:10.18632/oncotarget.5845)
Supplement: Supplementary file 2 [file oncotarget-06-40588-s002.docx]

**Supplementary Table 2.** The 285 probes corresponding to 128 unique genes that were differentially methylated in samples from patients with known and suspected clonal eosinophilia (S samples) and patients with reactive eosinophilia (R samples). The table is sorted by gene name.

| Probe ID | Chromosome | Cytosine position at chromosome | Neighboring gene* | Regulatory site annotated to probe | CpG neighborhood** | Δ methylation R vs. S |
| --- | --- | --- | --- | --- | --- | --- |
| cg03623568 | chr16 | 6915990 | *A2BP1* | 5'UTR | Open | -0,378246028 |
| cg10523679 | chr1 | 76189770 | *ACADM* | TSS1500 | Shore | -0,213186055 |
| cg13354988 | chr2 | 263656 | *ACP1* | TSS1500 | Island | -0,208004162 |
| cg18085176 | chr4 | 7939940 | *AFAP1* | 5'UTR | Shore | 0,258378343 |
| cg12870014 | chr12 | 110450643 | *ANKRD13A* | Body | Open | 0,309269014 |
| cg15165122 | chr2 | 71206291 | *ANKRD53* | Body | Island | 0,211295406 |
| cg15825321 | chr17 | 36666642 | *ARHGAP23* | Body | Island | 0,202564676 |
| cg13771313 | chr11 | 72533295 | *ATG16L2* | Body | Island | 0,31125968 |
| cg04006327 | chr11 | 72533487 | *ATG16L2* | Body | Island | 0,249988765 |
| cg16028117 | chr3 | 11348436 | *ATG7* | Body | Open | -0,219613565 |
| cg23159337 | chr3 | 193272778 | *ATP13A4* | TSS200 | Open | -0,216590562 |
| cg16744531 | chr19 | 17905626 | *B3GNT3* | TSS1500 | Island | 0,259775203 |
| cg13077366 | chr18 | 34908626 | *BRUNOL4* | Body | Open | 0,437276891 |
| cg23371584 | chr1 | 203275927 | *BTG2* | Body | Shore | 0,271259913 |
| cg02721176 | chr10 | 118084587 | *C10orf96* | Body | Open | -0,25683194 |
| cg24088508 | chr1 | 38156462 | *C1orf109* | TSS1500 | Shore | -0,264583885 |
| cg14279361 | chr19 | 6721955 | *C3* | TSS1500 | Open | 0,349232249 |
| cg02691506 | chr3 | 8685827 | *C3orf32* | 5'UTR | Open | 0,228349956 |
| cg25072766 | chr11 | 3073488 | *CARS* | Body | Open | 0,216712281 |
| cg17004353 | chr11 | 3073441 | *CARS* | Body | Open | 0,201452211 |
| cg03711050 | chr12 | 49297541 | *CCDC65* | TSS1500 | Open | 0,222107157 |
| cg24851651 | chr11 | 66362959 | *CCS* | Body | Shelf | 0,208491695 |
| cg19867917 | chr2 | 3642629 | *COLEC11* | TSS200 | Island | 0,206962772 |
| cg24732344 | chr7 | 137559892 | *CREB3L2* | 3'UTR | Open | -0,21172791 |
| cg04329125 | chr14 | 105945022 | *CRIP2* | Body | Island | 0,284525335 |
| cg27279301 | chr14 | 105945287 | *CRIP2* | Body | Island | 0,232035791 |
| cg25752163 | chr12 | 111470657 | *CUX2* | TSS1500 | Shore | 0,202267824 |
| cg04922029 | chr1 | 159174728 | *DARC* | TSS1500 | Open | 0,268206946 |
| cg12856521 | chr11 | 46389249 | *DGKZ* | Body | Island | 0,303569258 |
| cg05734675 | chr10 | 537033 | *DIP2C* | Body | Open | 0,246310267 |
| cg15930703 | chr2 | 225775142 | *DOCK10* | Body | Open | 0,231912855 |
| cg22047282 | chr2 | 27301649 | *EMILIN1* | 1stExon | Shelf | 0,214041616 |
| cg07608565 | chr19 | 55595022 | *EPS8L1* | Body | Island | 0,21781513 |
| cg05875700 | chr8 | 638208 | *ERICH1* | Body | Island | 0,241000427 |
| cg15909132 | chr17 | 42431109 | *FAM171A2* | Body | Island | 0,211892543 |
| cg15878909 | chr12 | 8380286 | *FAM90A1* | TSS200 | Open | 0,326375262 |
| cg09982942 | chr2 | 112898400 | *FBLN7* | Body | Shore | 0,209019784 |
| cg22361859 | chr17 | 37123638 | *FBXO47* | 1stExon | Open | -0,220210451 |
| cg22325292 | chr17 | 80708367 | *FN3K* | Body | Island | 0,272669468 |
| cg14500486 | chr6 | 159655392 | *FNDC1* | Body | Island | -0,23881544 |
| cg12542255 | chr19 | 45976195 | *FOSB* | Body | Island | 0,222713484 |
| cg22857963 | chr8 | 144358043 | *GLI4* | Body | Island | 0,355012048 |
| cg14825413 | chr8 | 144358566 | *GLI4* | Body | Island | 0,273077473 |
| cg09658497 | chr7 | 2847517 | *GNA12* | Body | Open | 0,233130618 |
| cg11630392 | chr3 | 150920964 | *GPR171* | 1stExon | Open | -0,249413363 |
| cg07380907 | chr16 | 11963779 | *GSPT1* | 3'UTR | Open | -0,206097864 |
| cg10950028 | chr1 | 110230633 | *GSTM1* | Body | Shore | -0,204344401 |
| cg11680055 | chr1 | 110230252 | *GSTM1* | TSS200 | Island | -0,251335231 |
| cg23719124 | chr1 | 110254919 | *GSTM5* | 1stExon | Open | -0,209816724 |
| cg22864244 | chr1 | 110255096 | *GSTM5* | Body | Open | -0,21935763 |
| cg25210835 | chr1 | 110254828 | *GSTM5* | TSS200 | Open | -0,247275941 |
| cg24467349 | chr1 | 110254835 | *GSTM5* | TSS200 | Open | -0,2588711 |
| cg20803293 | chr1 | 110254709 | *GSTM5* | TSS200 | Open | -0,293452218 |
| cg05376982 | chr1 | 110254692 | *GSTM5* | TSS200 | Open | -0,297856508 |
| cg25593510 | chr1 | 110254662 | *GSTM5* | TSS1500 | Open | -0,324999191 |
| cg07792871 | chr6 | 29942706 | *HCG9* | TSS200 | Shore | -0,244055213 |
| cg16672562 | chr19 | 46801672 | *HIF3A* | 5'UTR | Shore | 0,235701178 |
| cg22891070 | chr19 | 46801642 | *HIF3A* | Body | Shore | 0,230812856 |
| cg26038582 | chr1 | 42384390 | *HIVEP3* | 1stExon | Shore | 0,390901428 |
| cg25607920 | chr1 | 42384365 | *HIVEP3* | 1stExon | Shore | 0,372973573 |
| cg23762517 | chr1 | 42384310 | *HIVEP3* | 1stExon | Shore | 0,251651749 |
| cg05030953 | chr6 | 31241000 | *HLA-C* | TSS1500 | Shore | 0,307550818 |
| cg00620824 | chr6 | 31240784 | *HLA-C* | TSS1500 | Shore | 0,229682217 |
| cg17096289 | chr6 | 31238788 | *HLA-C* | Body | Shore | 0,209861045 |
| cg08269402 | chr6 | 32549631 | *HLA-DRB1* | Body | Shelf | -0,208880518 |
| cg12736254 | chr6 | 32557419 | *HLA-DRB1* | 1stExon | Open | -0,263192887 |
| cg11404906 | chr6 | 32551749 | *HLA-DRB1* | Body | Shore | -0,310091518 |
| cg23622369 | chr17 | 40706682 | *HSD17B1* | Body | Island | 0,264928855 |
| cg14089881 | chr1 | 6296630 | *ICMT* | TSS1500 | Shore | 0,248638755 |
| cg27485108 | chr2 | 26951130 | *KCNK3* | Body | Island | 0,232271459 |
| cg20418394 | chr10 | 72254335 | *KIAA1274* | 5'UTR | Open | 0,228462885 |
| cg06935979 | chr1 | 232941706 | *KIAA1383* | 1stExon | Island | 0,270252017 |
| cg00951395 | chr1 | 232941775 | *KIAA1383* | 1stExon | Shore | 0,214648569 |
| cg26347746 | chr1 | 202172848 | *LGR6* | TSS200 | Open | 0,318054972 |
| cg06825163 | chr1 | 202172912 | *LGR6* | 1stExon | Open | 0,305593498 |
| cg04811114 | chr1 | 202172778 | *LGR6* | TSS200 | Open | 0,304367369 |
| cg05044291 | chr1 | 202172867 | *LGR6* | TSS200 | Open | 0,242471025 |
| cg04234412 | chr22 | 24373322 | *LOC391322* | Body | Island | 0,201361049 |
| cg06975979 | chr7 | 150020025 | *LRRC61* | TSS1500 | Island | 0,208932047 |
| cg03185704 | chr7 | 150020125 | *LRRC61* | TSS200 | Island | 0,205132794 |
| cg03816081 | chr10 | 29577743 | *LYZL1* | TSS1500 | Open | 0,215581383 |
| cg05493407 | chr4 | 164816619 | *MARCH1* | 5'UTR | Open | 0,274473648 |
| cg23850205 | chr19 | 7967913 | *MAP2K7* | TSS1500 | Shore | 0,243183756 |
| cg01435643 | chr13 | 113689776 | *MCF2L;MCF2L* | Body | Shore | 0,227157569 |
| cg24796644 | chr6 | 37617956 | *MDGA1* | Body | Island | -0,252829353 |
| cg14926196 | chr6 | 37616482 | *MDGA1* | Body | Island | -0,263900901 |
| cg04737881 | chr4 | 183060943 | *MGC45800* | Body | Shore | 0,235137123 |
| cg26328633 | chr5 | 135416394 | *MIR886* | TSS200 | Island | 0,278428743 |
| cg25340688 | chr5 | 135416398 | *MIR886* | TSS200 | Island | 0,275770624 |
| cg06536614 | chr5 | 135416381 | *MIR886* | TSS200 | Island | 0,271288044 |
| cg00124993 | chr5 | 135416412 | *MIR886* | TSS200 | Island | 0,24955566 |
| cg18797653 | chr5 | 135416613 | *MIR886* | TSS1500 | Shore | 0,232853871 |
| cg16615357 | chr5 | 135416594 | *MIR886* | TSS1500 | Shore | 0,221580444 |
| cg26896946 | chr5 | 135416405 | *MIR886* | TSS200 | Island | 0,221458068 |
| cg18678645 | chr5 | 135416331 | *MIR886* | TSS200 | Island | 0,214210799 |
| cg04481923 | chr5 | 135416205 | *MIR886* | Body | Island | 0,20503005 |
| cg18315834 | chr14 | 64909119 | *MTHFD1* | Body | Open | -0,21591586 |
| cg00414384 | chr11 | 68518048 | *MTL5* | Body | Island | 0,238054557 |
| cg08257257 | chr11 | 68517804 | *MTL5;MTL5* | Body | Island | 0,22881082 |
| cg03329597 | chr3 | 108125523 | *MYH15* | Body | Open | -0,396507017 |
| cg16077055 | chr2 | 106428750 | *NCK2* | 5'UTR | Open | -0,20217579 |
| cg03904042 | chr20 | 32255491 | *NECAB3* | Body | Island | 0,304278985 |
| cg20976694 | chr18 | 55714052 | *NEDD4L* | Body | Shore | -0,261266978 |
| cg12863693 | chr15 | 85201151 | *NMB* | Body | Shore | 0,252896511 |
| cg11787167 | chr14 | 33407370 | *NPAS3* | TSS1500 | Shelf | 0,241085676 |
| cg00404641 | chr3 | 131080516 | *NUDT16P* | TSS200 | Shore | 0,26850736 |
| cg22575379 | chr3 | 131080235 | *NUDT16P* | TSS1500 | Shore | 0,216599767 |
| cg14230378 | chr3 | 131080483 | *NUDT16P* | TSS1500 | Shore | 0,214680314 |
| cg13725599 | chr20 | 3052262 | *OXT* | TSS200 | Island | 0,23713434 |
| cg16887334 | chr20 | 3052151 | *OXT* | TSS200 | Island | 0,234299677 |
| cg24996161 | chr5 | 140735397 | *PCDHGA2* | Body | Shore | -0,220057807 |
| cg10864200 | chr4 | 720809 | *PCGF3* | 5'UTR | Shelf | 0,293707742 |
| cg14465747 | chr7 | 32111342 | *PDE1C* | TSS1500 | Shore | -0,244308882 |
| cg15398841 | chr4 | 74847761 | *PF4* | TSS200 | Island | 0,201301215 |
| cg01412970 | chr17 | 17109239 | *PLD6* | 1stExon | Island | 0,279042556 |
| cg26906998 | chr17 | 17108846 | *PLD6* | Body | Island | 0,221528532 |
| cg22445712 | chr22 | 50738550 | *PLXNB2* | 5'UTR | Island | 0,226411896 |
| cg26354017 | chr1 | 205819088 | *PM20D1* | 1stExon | Island | -0,207958903 |
| cg17178900 | chr1 | 205818956 | *PM20D1* | Body | Island | -0,246957642 |
| cg19236675 | chr7 | 76624761 | *PMS2L11* | Body | Open | 0,421901685 |
| cg07516511 | chr16 | 2907819 | *PRSS22* | Body | Island | 0,287802495 |
| cg09745688 | chr16 | 2908918 | *PRSS22* | TSS1500 | Shore | 0,264163645 |
| cg05895034 | chr16 | 2908892 | *PRSS22* | TSS1500 | Shore | 0,216310412 |
| cg11708721 | chr1 | 117530009 | *PTGFRN* | 3'UTR | Open | 0,237511384 |
| cg24687529 | chr1 | 117529954 | *PTGFRN* | 3'UTR | Open | 0,216561781 |
| cg02527881 | chr3 | 46936655 | *PTH1R* | Body | Shelf | 0,208831444 |
| cg23299919 | chr7 | 157406096 | *PTPRN2* | Body | Island | 0,210104342 |
| cg26786045 | chr11 | 61672207 | *RAB3IL1* | Body | Open | 0,303096484 |
| cg10140454 | chr17 | 17626019 | *RAI1* | 5'UTR | Island | 0,248674706 |
| cg27509823 | chr1 | 173930183 | *RC3H1* | Body | Open | -0,332445466 |
| cg12062782 | chr11 | 66102686 | *RIN1* | Body | Shore | 0,246638287 |
| cg03226844 | chr17 | 185102 | *RPH3AL* | 5'UTR | Island | 0,214154783 |
| cg03329755 | chr6 | 167189272 | *RPS6KA2* | Body | Open | -0,20066674 |
| cg09508496 | chr19 | 39056084 | *RYR1* | Body | Island | 0,233524986 |
| cg02452418 | chr19 | 39056126 | *RYR1* | Body | Island | 0,207458107 |
| cg11343894 | chr1 | 153599704 | *S100A13* | 5'UTR | Open | 0,241327368 |
| cg02331910 | chr1 | 153599831 | *S100A13* | 5'UTR | Open | 0,227929191 |
| cg13946767 | chr1 | 153599671 | *S100A13* | 5'UTR | Open | 0,222833112 |
| cg06419659 | chr1 | 153600132 | *S100A13* | TSS1500 | Open | 0,205494044 |
| cg24441899 | chr7 | 4244372 | *SDK1* | Body | Open | -0,414145685 |
| cg16141740 | chr22 | 42896985 | *SERHL* | Body | Island | 0,230277402 |
| cg04505435 | chr11 | 70672511 | *SHANK2* | Body | Shore | 0,251151371 |
| cg25456593 | chr11 | 70672858 | *SHANK2* | Body | Island | 0,2205842 |
| cg11480627 | chr11 | 70672876 | *SHANK2* | Body | Island | 0,205524196 |
| cg18357371 | chr6 | 25874590 | *SLC17A3* | TSS200 | Open | 0,216353626 |
| cg14228592 | chr8 | 145639181 | *SLC39A4* | Body | Island | 0,312527743 |
| cg02583091 | chr8 | 145638881 | *SLC39A4* | Body | Island | 0,244515305 |
| cg05681977 | chr8 | 145638934 | *SLC39A4* | Body | Island | 0,243131851 |
| cg05967295 | chr7 | 98741636 | *SMURF1* | 1stExon | Island | 0,204386161 |
| cg04392082 | chr4 | 186732926 | *SORBS2* | 5'UTR | Open | 0,230504681 |
| cg01933073 | chr4 | 186732942 | *SORBS2* | 5'UTR | Open | 0,215779851 |
| cg22138735 | chr20 | 62679713 | *SOX18* | Body | Island | 0,295323631 |
| cg09549987 | chr8 | 7320532 | *SPAG11B* | Body | Open | -0,527933404 |
| cg01286930 | chr2 | 54783773 | *SPTBN1* | Body | Shore | 0,225280044 |
| cg18872420 | chr14 | 78023429 | *SPTLC2* | Body | Open | -0,266223117 |
| cg25851277 | chr3 | 42631429 | *SS18L2* | TSS1500 | Shore | -0,228388392 |
| cg02398342 | chr17 | 80708632 | *TBCD* | TSS1500 | Shore | 0,265154375 |
| cg00159523 | chr10 | 114713187 | *TCF7L2* | Body | Shore | 0,244724921 |
| cg11961237 | chr12 | 111052267 | *TCTN1* | Body | Shore | 0,220387842 |
| cg01542019 | chr19 | 14673053 | *TECR* | Body | Shelf | 0,233257346 |
| cg06259025 | chr13 | 52769875 | *THSD1P* | TSS1500 | Island | 0,201793346 |
| cg07620230 | chr20 | 62328084 | *TNFRSF6B* | 5'UTR | Island | 0,279073749 |
| cg24354818 | chr20 | 62328094 | *TNFRSF6B* | 5'UTR | Island | 0,270902518 |
| cg16702083 | chr20 | 62328427 | *TNFRSF6B* | Body | Island | 0,268432039 |
| cg07524919 | chr6 | 32063901 | *TNXB* | Body | Island | 0,280489426 |
| cg01992382 | chr6 | 32064212 | *TNXB* | Body | Island | 0,259281558 |
| cg10890302 | chr6 | 32064246 | *TNXB* | Body | Island | 0,257556225 |
| cg00872984 | chr6 | 32063991 | *TNXB* | Body | Island | 0,248186694 |
| cg27387193 | chr6 | 32064032 | *TNXB* | Body | Island | 0,235426251 |
| cg10923662 | chr6 | 32064258 | *TNXB* | Body | Island | 0,232698222 |
| cg00525277 | chr6 | 32064239 | *TNXB* | Body | Island | 0,200690633 |
| cg04498198 | chr17 | 27899966 | *TP53I13* | 3'UTR | Island | 0,214282301 |
| cg19843353 | chr5 | 180651585 | *TRIM41* | 1stExon | Shore | 0,204490621 |
| cg17901382 | chr17 | 73514798 | *TSEN54* | Body | Shore | 0,267496269 |
| cg26744682 | chr19 | 50249584 | *TSKS* | Body | Island | -0,268020892 |
| cg14709479 | chr1 | 1108924 | *TTLL10* | TSS1500 | Shore | 0,232372626 |
| cg08441918 | chr19 | 6495210 | *TUBB4* | Body | Island | 0,267254697 |
| cg01514075 | chr1 | 7832357 | *VAMP3* | Body | Shore | -0,244845686 |
| cg01718139 | chr19 | 54566838 | *VSTM1* | Body | Open | 0,203592892 |
| cg21330207 | chr18 | 72916311 | *ZADH2* | Body | Island | 0,361733328 |
| cg02750262 | chr18 | 72916776 | *ZADH2* | Body | Island | 0,307435007 |
| cg03972071 | chr18 | 72917163 | *ZADH2* | Body | Island | 0,290491708 |
| cg11568697 | chr18 | 72916393 | *ZADH2* | Body | Island | 0,22331582 |
| cg21786191 | chr18 | 72916012 | *ZADH2* | Body | Shore | 0,207262074 |
| cg20744163 | chr10 | 80999841 | *ZMIZ1* | Body | Shelf | 0,282896322 |
| cg01615818 | chr19 | 23941438 | *ZNF681* | Body | Island | 0,249892007 |
| cg08332658 | chr19 | 23941408 | *ZNF681* | Body | Island | 0,213734635 |
| cg07460376 | chr19 | 23941575 | *ZNF681* | 1stExon | Shore | 0,207942784 |
| cg05388281 | chr4 | 125504 | *ZNF718* | Body | Shore | 0,247945901 |
| cg12717203 | chr4 | 124342 | *ZNF718* | Body | Island | 0,227620683 |
| cg20320494 | chr4 | 124232 | *ZNF718* | Body | Shore | 0,207253018 |
| cg08291996 | chr19 | 58791159 | *ZNF8* | Body | Shore | 0,303540579 |
| cg02631126 | chr6 | 28058918 | *ZSCAN12L1* | TSS200 | Open | -0,201835269 |
| cg27665985 | chr15 | 66116727 | - | - | Open | 2,735549701 |
| cg24033633 | chr3 | 57945695 | - | - | Open | 0,396259857 |
| cg15052665 | chr6 | 141804349 | - | - | Open | 0,353751086 |
| cg25987564 | chr4 | 6010075 | - | - | Island | 0,318521171 |
| cg03447554 | chr11 | 43094025 | - | - | Open | 0,303506964 |
| cg10090844 | chr12 | 132167226 | - | - | Shelf | 0,303452265 |
| cg03582285 | chr17 | 40700314 | - | - | Island | 0,28915566 |
| cg14602393 | chr12 | 133343405 | - | - | Shelf | 0,287182917 |
| cg03738707 | chr2 | 128453335 | - | - | Island | 0,285662563 |
| cg23019889 | chr1 | 146544044 | - | - | Island | 0,284393331 |
| cg16462006 | chr14 | 95155784 | - | - | Open | 0,28125734 |
| cg18522231 | chr3 | 196705629 | - | - | Open | 0,276822515 |
| cg23549902 | chr7 | 5184155 | - | - | Island | 0,273889267 |
| cg01219135 | chr7 | 158766336 | - | - | Island | 0,272489832 |
| cg07123855 | chr22 | 39323204 | - | - | Island | 0,271053008 |
| cg23758822 | chr17 | 41437982 | - | - | Shore | 0,269800681 |
| cg16824113 | chr12 | 132166485 | - | - | Open | 0,268058462 |
| cg17172308 | chr6 | 168533631 | - | - | Shelf | 0,267651753 |
| cg08767686 | chr5 | 51426 | - | - | Island | 0,261911893 |
| cg02159489 | chr17 | 79459563 | - | - | Shelf | 0,256933499 |
| cg05856321 | chr17 | 1133546 | - | - | Island | 0,255441079 |
| cg18025438 | chr1 | 228756789 | - | - | Island | 0,254204654 |
| cg05422022 | chr6 | 168533689 | - | - | Shelf | 0,25242893 |
| cg01166350 | chr14 | 64228624 | - | - | Open | 0,251353728 |
| cg01760090 | chr8 | 1365659 | - | - | Shore | 0,249737387 |
| cg21513088 | chr2 | 585365 | - | - | Shore | 0,247384753 |
| cg12744031 | chr7 | 158751184 | - | - | Island | 0,246584968 |
| cg02270332 | chr6 | 106475062 | - | - | Open | 0,246578231 |
| cg09590377 | chr2 | 8597389 | - | - | Island | 0,246009922 |
| cg04048131 | chr13 | 107031945 | - | - | Open | 0,245610096 |
| cg08474748 | chr5 | 74350214 | - | - | Island | 0,244643792 |
| cg03393996 | chr4 | 1580377 | - | - | Island | 0,243801682 |
| cg19183395 | chr2 | 233216865 | - | - | Island | 0,243008007 |
| cg02291365 | chr13 | 25317267 | - | - | Shelf | 0,238976527 |
| cg03701930 | chr10 | 1981436 | - | - | Open | 0,237452306 |
| cg25573640 | chr14 | 64228599 | - | - | Open | 0,237315918 |
| cg19313311 | chr7 | 74058031 | - | - | Open | 0,236365815 |
| cg03396347 | chr1 | 1875803 | - | - | Island | 0,2337164 |
| cg10486069 | chr11 | 133658113 | - | - | Open | 0,231591124 |
| cg01701555 | chr1 | 146544097 | - | - | Island | 0,231376478 |
| cg25277809 | chr2 | 128453484 | - | - | Shore | 0,230584831 |
| cg17794299 | chr20 | 623187 | - | - | Island | 0,23008166 |
| cg23733394 | chr1 | 839752 | - | - | Island | 0,225709332 |
| cg05547895 | chr3 | 196705855 | - | - | Open | 0,224991237 |
| cg25298319 | chr2 | 233217079 | - | - | Island | 0,223869054 |
| cg07158503 | chr5 | 135415693 | - | - | Shore | 0,223634539 |
| cg17856063 | chr1 | 146543992 | - | - | Island | 0,221926632 |
| cg25731401 | chr2 | 10572392 | - | - | Island | 0,221623438 |
| cg17392866 | chr15 | 32267708 | - | - | Open | 0,221238686 |
| cg11608150 | chr5 | 135415948 | - | - | Shore | 0,220543005 |
| cg21434132 | chr3 | 196705742 | - | - | Open | 0,219613005 |
| cg10225865 | chr8 | 54605566 | - | - | Open | 0,219299095 |
| cg25960378 | chr2 | 228190026 | - | - | Island | 0,219157965 |
| cg06777732 | chr12 | 131118426 | - | - | Open | 0,219120559 |
| cg04090468 | chr7 | 156181990 | - | - | Open | 0,21905516 |
| cg07961098 | chr14 | 64228680 | - | - | Open | 0,218411983 |
| cg00413089 | chr7 | 158750985 | - | - | Island | 0,217135719 |
| cg08164151 | chr12 | 131118432 | - | - | Open | 0,215206886 |
| cg00748494 | chr13 | 23412343 | - | - | Island | 0,215193844 |
| cg15910301 | chr14 | 71632612 | - | - | Open | 0,215147778 |
| cg18826637 | chr2 | 145116633 | - | - | Open | 0,214070537 |
| cg10760651 | chr1 | 86968184 | - | - | Open | 0,209402308 |
| cg25691825 | chr18 | 74499511 | - | - | Island | 0,208284461 |
| cg14882966 | chr2 | 3699353 | - | - | Island | 0,206332576 |
| cg14395298 | chr13 | 23412250 | - | - | Island | 0,206120943 |
| cg16548362 | chr15 | 101389394 | - | - | Shore | 0,205706156 |
| cg07308384 | chr6 | 114697971 | - | - | Open | 0,205606453 |
| cg15572235 | chr7 | 5183992 | - | - | Island | 0,204966037 |
| cg07694864 | chr17 | 40700509 | - | - | Island | 0,204775267 |
| cg21333674 | chr8 | 96705765 | - | - | Open | 0,204169231 |
| cg03996861 | chr8 | 54605613 | - | - | Open | 0,20377798 |
| cg26064774 | chr4 | 6666009 | - | - | Island | 0,201848151 |
| cg05250119 | chr15 | 79123384 | - | - | Island | 0,200799826 |
| cg23420331 | chr2 | 228190042 | - | - | Island | 0,200686098 |
| cg04645160 | chr15 | 96913183 | - | - | Shore | 0,200071335 |
| cg08040755 | chr14 | 76716894 | - | - | Open | -0,203098275 |
| cg03680873 | chr3 | 148844300 | - | - | Shelf | -0,203504287 |
| cg10742917 | chr7 | 56242801 | - | - | Island | -0,203696549 |
| cg23250574 | chr6 | 28661310 | - | - | Open | -0,204634367 |
| cg08817483 | chr17 | 20744547 | - | - | Island | -0,206001552 |
| cg25308322 | chr13 | 86268291 | - | - | Open | -0,206628248 |
| cg25428263 | chr17 | 34599556 | - | - | Shore | -0,207294084 |
| cg02591356 | chr2 | 20442088 | - | - | Open | -0,212531078 |
| cg01091514 | chr4 | 184644613 | - | - | Shore | -0,231354547 |
| cg20909645 | chr8 | 99985049 | - | - | Island | -0,231464184 |
| cg23549331 | chr8 | 6189008 | - | - | Open | -0,235939952 |
| cg02487331 | chr1 | 146550467 | - | - | Island | -0,236375589 |
| cg12133451 | chr1 | 227746453 | - | - | Island | -0,251560809 |
| cg03526459 | chr1 | 146549940 | - | - | Island | -0,270535554 |
| cg00698771 | chr1 | 113285940 | - | - | Shore | -0,309526941 |
| cg05528899 | chr17 | 57120 | - | - | Island | -0,321825118 |
| cg21139150 | chr21 | 46976175 | - | - | Island | -0,348779234 |

* - Intergenic probes

** Shore: 0-2 Kb from nearest CpG Island; Shelf: 2-4 Kb from CpG Island; Open: Non CpG
